# Young adult cancer survivors’ experience of taking part in a 12-week exercise referral programme: a qualitative study of the Trekstock RENEW initiative

**DOI:** 10.1007/s00520-020-05746-w

**Published:** 2020-09-22

**Authors:** N. Below, A. Fisher, S. Epstone, J. Reynolds, G Pugh

**Affiliations:** 1 Centre for Sports & Exercise Medicine, Barts & The London School of Medicine, Queen Mary University of London, Mile End Hospital, London, UK; 2 Department of Behavioural Science & Health, University College London, London, UK; 3 Trekstock, London, UK

**Keywords:** Physical activity, Exercise programme, Young adult cancer survivors, Qualitative evaluation, Adherence, Compliance, Barriers

## Abstract

**Purpose:**

There is emerging evidence that physical activity interventions have the potential to improve the physical function and psychosocial well-being of young adult cancer survivors. However, most existing interventions for young adult cancer survivors have been delivered in an in-patient hospital setting. The purpose of this study is to explore young adult cancer survivors’ (YACS) experiences of the RENEW programme, a 12-week community-based exercise referral scheme delivered by Trekstock, a UK-based cancer charity.

**Methods:**

Sixteen semi-structured interviews were conducted with YACS (mean age, 33 years; 87.5% female) who participated in the RENEW exercise referral programme. Each interview followed the same semi-structured interview guide which asked participants about their experiences of the RENEW programme and their ideas for the future development of the scheme. Data was audio-recorded, transcribed full verbatim and analysed using framework analysis.

**Results:**

YACs predominantly chose to take part in the RENEW programme as a means to improve their health and overcome cancer related impairments (e.g. fatigue, loss of strength, low body confidence). The offer of one-to-one tailored support and unlimited gym access was often cited as a factor which motivated enrolment. Overall, YACS experience of the programme was positive with many describing improvements in physical function and general well-being. Barriers to participating in the programme included sign-off from clinicians prior to enrolment, travelling to the gym and fear of exercising alone.

**Conclusions:**

Exercise referral schemes are acceptable to YACS and provide a promising opportunity for young people with cancer to improve their physical and psychosocial health through physical activity.

**Electronic supplementary material:**

The online version of this article (10.1007/s00520-020-05746-w) contains supplementary material, which is available to authorized users.

## Introduction

Each day 34 young adults are diagnosed with cancer in the UK [1]. Due to improvements in treatment and care, the 5-year overall survival rate among this age group is around 80% [2]. However, intensive treatment regimes, treatment-related side effects and prolonged periods isolated in hospital or at home away from higher education, work and peer groups can compromise young adult cancer survivors’ physical and psychosocial well-being [3–5]. There is growing evidence that physical activity is an effective non-pharmacological intervention to remediate the ongoing effects of cancer and its treatment, such as fatigue and declines in physical fitness [6]. However, the majority of research thus far has been conducted in controlled clinical environments among cancer survivors over the age of 50 diagnosed with breast, prostate or colorectal cancer [7–9].

At present there are very few evidence-based physical activity programmes designed specifically for young adult cancer survivors. Most intervention studies focus upon either children and adolescents undergoing treatment or young adult survivors of childhood cancer [10]. In addition, most community-based physical activity interventions for cancer survivors are tailored towards older adults [11]. To address this gap in care, Trekstock (a London-based charity which provides support programmes for young adults with cancer) developed a 12-week exercise referral programme titled RENEW. The purpose of the RENEW programme was to address young adult cancer survivors’ need and desire for structured physical activity support [12]. The RENEW programme provided personal training support from a qualified Level 4 Cancer Rehabilitation instructor within a community-based gym setting, free gym membership and access to online support materials. An evaluation of the RENEW programme indicated that this intervention improved young adult cancer survivors’ levels of physical activity, physical function, fatigue, sleep and health-related quality of life [13].

Although the RENEW programme demonstrates positive impact, the varying level of compliance with the programme and homogenous sample of participants suggest a need to further explore the factors underpinning intervention engagement. Understanding patient experience is vital to inform the design and implementation of new services, particularly to ensure maximum access, uptake, adherence and benefit [14]. Qualitative methodologies are recognised as an effective way to capture the patient voice and allow in-depth understanding to inform intervention and service improvement in cancer care [15].

The aim of this study was therefore to explore young people’s experiences of the 12-week RENEW exercise programme and to establish their ideas for the future development of the programme.

## Methods

### Participants and recruitment

Participants were eligible to take part in the RENEW programme if they were aged 20–39 and had received a cancer diagnosis at any point in their lifetime. Data on recruitment and attrition to the RENEW programme is reported elsewhere [13]. An invitation to participate within this qualitative study was distributed by the Health Programmes Lead at Trekstock in July 2019 through email invitations and advertisements shared upon social media. All RENEW participants (*n* = 76) were invited to take part in the interview study regardless of whether they had completed the full 12 weeks of the programme.

### Ethical approval

Ethical approval for this study was provided by Queen Mary University Research Ethics Committee (reference: QMREC2018/48/006), and all participants gave informed written consent. No incentive or compensation was provided to participants taking part in the study.

### Qualitative interviews

In July and August 2019, semi-structured telephone interviews were conducted by one researcher (NB, female medical student with no prior relationship with participants). All participants were provided with an information sheet explaining the purpose of the study and were asked to provide informed written consent before taking part. Each interview followed the same interview guide (Supplementary File A) which covered (i) participants’ motives for taking part in the RENEW programme, (ii) expectations of the RENEW programme, (iii) overall satisfaction and experience of the programme, (iv) any challenges faced during the programme and (v) their ideas for how the RENEW programme could be improved. A variety of probes were used when necessary, and participants were encouraged to share their thoughts and experiences openly. The interview process was audio-recorded on a dictaphone, anonymised and transcribed full verbatim before analysis. Field notes were taken throughout each interview.

### Analysis

**Fig. 1:**
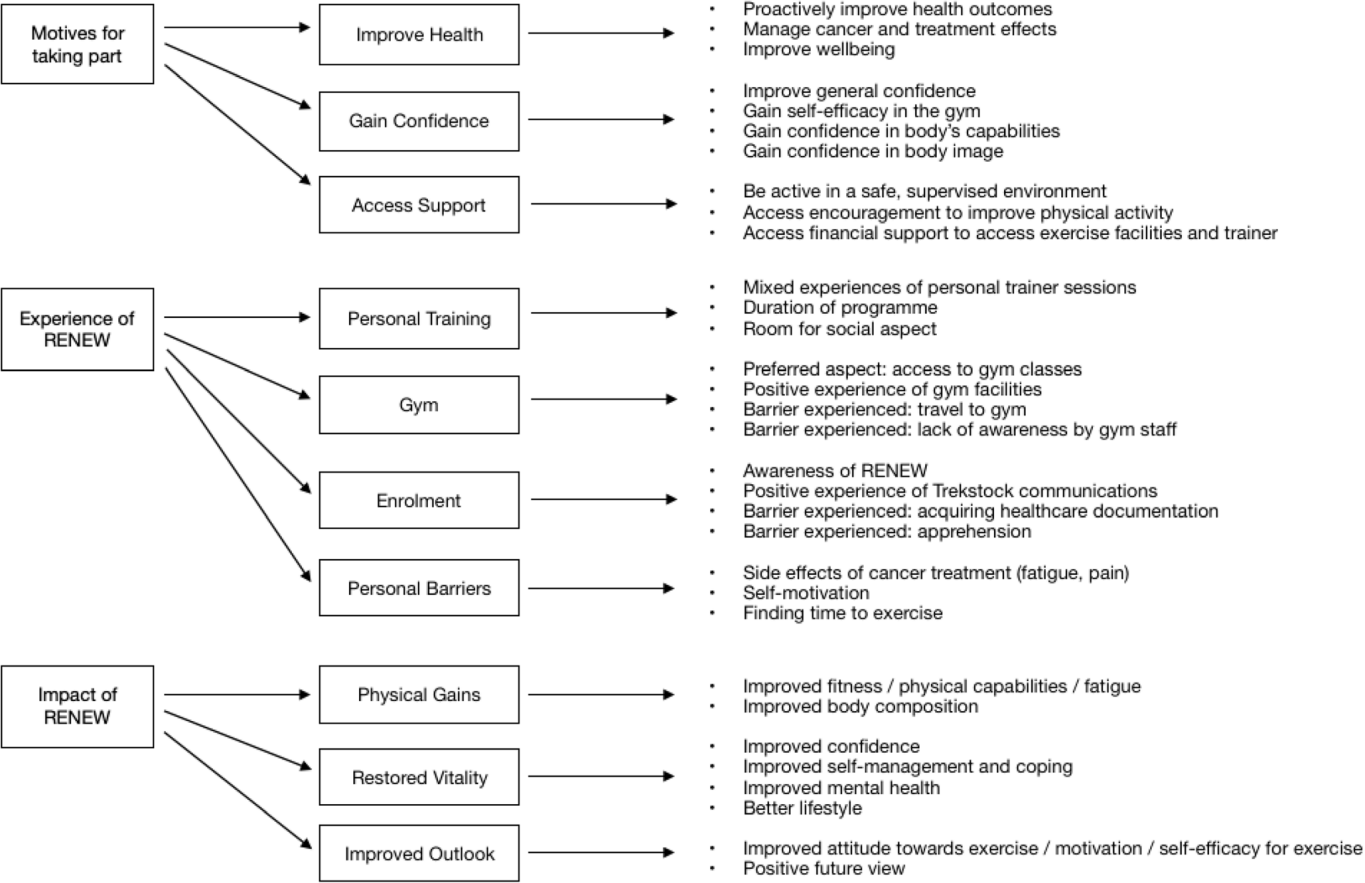
Themes and sub-themes

Framework analysis was used following the protocol outlined by Gale and colleagues [16]. Researchers NB and GP independently analysed three transcripts, each developing an initial set of codes. The researchers then compared these, making minor adjustments to create the final analytical framework, which was used to code the remaining transcripts. Participants’ responses were summarised and coded into a framework matrix in Microsoft Excel. NB and GP independently interpreted the data to identify common themes among responses and then met to discuss their interpretations. Key themes and quotes were collated and summarised to create the final thematic map (Fig. 1).

## Results

Sixteen of the 76 participants who took part in the RENEW programme chose to participate within the interview study (response rate, 21%). Table 1 displays participant characteristics. Participants were aged between 22 and 39 years (mean age 33 years); most had completed active cancer treatment (*n* = 14, 87.5%) and were female (*n* = 14, 87.5%). Participants were survivors of eight different cancer types, with the most prevalent types being breast and bowel. Interviews lasted on average 27.3 min (range 15–45 min).

**Table 1:** Characteristics of participants

Age, [range], (years)	32.6 [22-39]
Gender	
Female	14 (87.5%)
Male	2 (12.5%)
Cancer type	
Breast	6 (37.5%)
Bowel	4 (25%)
Dysgerminoma (ovarian)	1 (6.25%)
Womb	1 (6.25%)
Brain	1 (6.25%)
Kidney (Wilms’ tumour) + lung	1 (6.25%)
Non-Hodgkin’s lymphoma	1 (6.25%)
Acute myeloid leukaemia	1 (6.25%)
Treatment status	
Completed active treatment	14 (87.5%)
On active treatment	2 (12.5%)
Programme participation	
Completed	12 (75%)
Dropped out	2 (12.5%)
In programme	2 (12.5%)
Time since programme	
n/a (still in programme)	2 (12.5%)
< 1 month	2 (12.5%)
1–6 months	6 (37.5%)
7–12 months	5 (31.25%)
13–18 months	1 (6.25%)

Three major themes emerged: motives for taking part, experience of RENEW and impact of RENEW. Across these themes, ten subthemes emerged from the data with regard to the participant experience of the RENEW programme. These themes and sub-themes are illustrated in Fig. 1.

### Motivation for taking part

#### Health

Participants often disclosed that their primary motivation for taking part in the RENEW programme was to take ownership over their health; ‘to feel like I was actually doing something to help myself’ (F, 41)*.* Many participants felt exercise was an opportunity to restore their vitality, improve their capacity to carry out everyday tasks and cope with future illness.

At one point I couldn’t stand up long enough to brush my teeth without my legs hurting … when I heard about the RENEW that’s why I thought I needed to do that because at that point I was struggling just with life skills. (F,34)

#### Confidence

The opportunity to participate in the RENEW programme was also viewed as a means to restore confidence and self-efficacy to exercise.

I had no energy, I felt like I’d lost all my muscle. I couldn’t do anything. When I heard about the RENEW I was just, this is exactly what I need to do. I need help to just restart me, basically …I just didn’t have any confidence in my body. I thought I was probably going to faint or something. (34, F)

Participants described hoping to gain the confidence to use a gym without feeling stigmatised as a cancer patient, explaining that before RENEW, they felt ‘self-conscious’ (F, 37) about using gyms and wanted ‘a transition into being able to go into gyms without that feeling of nervousness because I can’t do as much as most young people’ (F, 26).

#### Support

Participants often described the appeal of individual advice from qualified instructors as being a key motivation behind signing up to the programme. Many participants described that they ‘didn’t really know where to start’ (F, 37) and wanted to ‘learn what was safe for me to do, learn how to do it safely, learn how often is a good idea, how to build up’ (F, 39). Several participants explained that due to the financial burden of cancer, they would not have been able to access one-to-one personal support without the aid of Trekstock.

### Experience of taking part

#### Personal training

Participants appreciated the opportunity to work with a personal trainer to ‘find ways [to exercise] that work for me’ (F, 39) with someone ‘who can just push you a little bit and tell you when you’re fine’ (F, 34). The associated accountability was identified as motivational, with participants feeling that they ‘had to have achieved something by the time [they] saw [the personal trainer] next’ (F, 34). Another advantage of the one-to-one structure of the programme was that each session could be personally tailored and structured to suit participants’ individual needs.

I’ve got certain problems which I couldn’t always get addressed in a group situation, so having one-to-one really made a difference. (F, 39)

The use of objective measurements was said to be motivational and a useful tool for monitoring progress and helped participants feel proud of themselves at the end of the 12 weeks.

It was nice to see the progress. … My weight on the scales wasn’t changing and that was quite demoralising but learning that you are losing fat and turning it into muscle, which I wouldn’t have known really, if I hadn’t done the RENEW thing, that was really good for me and it spurred me on to keep going. (F, 34)

#### Gym

Having access to the gym facilities and exercise classes was often described as a highlight. However, many participants found travelling to the gym a logistical barrier, and some felt attending the gym alone was intimidating. Participants suggested the expansion of the programme to other locations could be facilitated by partnerships with commercial gyms, and the introduction of a buddy system may encourage those who are apprehensive to attend the program.

Although [using] the gym itself was free, travelling in and out of London is £10 a time. So I was a bit limited. (F, 34)

I don’t know if you can work with any of the big chains so that you can use more local gyms, even if you had to go further for a PT, that would have been one thing that would have improved it most. (F, 34)

#### Barriers to enrolment and engagement

When asked about any challenges faced during the programme, many participants highlighted that side effects of cancer treatment (including pain and fatigue), self-motivation to exercise and time pressures limited their ability to engage with the programme fully.

I had a bit of time where my medication that I have to be on was causing me a lot of problems with pain and fatigue which got in the way of really me being able to go, but I had one of my one-on-one sessions that I got myself out for and I explained to the trainer and they were able to make adaptations to the exercises and give me suggestions for those for when the pain and fatigue was really bad, so I was still able to do something and even suggestions for what to do at home. It was too bad to get out, but not too bad that I couldn’t do something. (F, 26)

Some participants discussed apprehension and anxiety towards initially enrolling into the programme citing worry about their previous levels of physical activity and current physical function as barriers to signing up. However, these initial feelings were overcome by support from Trekstock and visual marketing of the RENEW programme on social media.

I was a bit nervous of having someone be there watching me do things and if I couldn’t manage it I’d feel silly or I’d feel let down with myself … it was just apprehension really, a bit anxious about it. (F, 38)

The emails I got from Trekstock when I was asking about it as well, they were lovely and reassuring as well, so that helped. (F, 38)

Another barrier to enrolment was acquiring healthcare documentation, as participants who were on active cancer treatment at the time of the programme were required to obtain a consent letter from their medical team. The main difficulty experienced was arranging appointments with doctors and the physical task of getting to the hospital or GP centre.

I think the most difficult thing was getting the letter signed by my GP because I gave it in and they said it was ready to collect but that was just after my surgery and I couldn’t even walk to my GP, I kind of walked half the way and then I sat down and I was like I just can’t go all the way so I walked back home. (F, 34)

It was quite hard to get my GP to do the sign-off as well. Round about that time I wasn’t seeing any hospital consultants. My hospital is quite a trek away, so it’s not like I could just pop in and get it done. (F, 38)

### Impact

The vast majority of participants described how the RENEW programme had dramatically improved their physical function and vitality. Most pertinently, participants described changes in activities of daily living which contributed to a regained sense of independence and purpose.

At the beginning she was like, “Do a five-minute walk on the treadmill,” and I was massively out of breath by the time I’d finished it and then by the end I was doing planks for a minute and squats like I used to do before all of this happened ... I was seeing things that I really wanted to get back to and I did a spinning class towards the end of the 12 weeks which took me to build up the courage to go, which is what I used to do a lot of and I was very out of breath, but I got through the whole 45 minute class. ... That felt like a huge milestone to be able to have the stamina to do that. (F, 35)

After having spent how many years of feeling terrible, it’s nice to be able to go “I can actually do some stuff for myself”. (F, 39)

They sort of go hand in hand, the more you feel better and stronger and like you’ve got no limitations, the better you feel and more you just can get back to normal life basically. (F, 35)

I see myself much less as a vulnerable, sick, cancer patient now. I don’t present myself in that way now and I think it definitely helped with that. (F, 37)

Participants also highlighted that the RENEW programme changed their relationship to physical activity and improved their understanding of the types of exercise they could do post cancer. Many participants noted that with the support of the RENEW programme, they were able to make exercise part of their everyday routine. There was a general consensus among participants that the RENEW programme had assisted them to move forward past their cancer diagnosis and regain a sense of normality.

I can sort of feel that I’m a gym user, yes, and that’s something that I can do which I didn’t know or didn’t feel that I could do before. … Because when you are recovering from something you miss out on lots of stuff, so it’s nice to be able to pick some of them back up. (M, 28)

Feeling your body getting better helped me to feel like okay I’m moving forward, when I keep moving forward from cancer. (F, 35)

## Discussion

The RENEW programme delivered by Trekstock was developed in order to provide young adult cancer survivors access to one-to-one physical activity support. Previous findings from the quantitative evaluation suggest the RENEW programme supported young adults with cancer to regain physical and mental strength. Findings from within this study indicate this was facilitated by the cancer-specific, individualised and cost-free aspects of the programme, which helped participants to overcome personal barriers relating to motivation and side effects of cancer treatment. These findings are fundamental to understanding the meaningfulness of exercise-based supportive care interventions from the perspectives of young adults with cancer.

Cancer and its associated treatment can have a huge effect on survivors’ physical and psychosocial well-being. Many participants within this study disclosed that they specifically enrolled into the RENEW programme to improve their health and address cancer-related impairments such as fatigue and loss of strength. Several existing studies confirm these findings that health behaviour (specifically physical activity) is often used by cancer survivors as a self-management strategy to proactively take ownership of their health [17, 18]. Similarly a meta-synthesis of qualitative studies on cancer survivors’ experience of participation in exercise-based rehabilitation identified affirmation of own health and new purpose as key themes across studies [19]. Physical activity provides a means for individuals to regain confidence and self-efficacy supporting a shift away from the sick, ill, or frail narrative which accompanies a cancer diagnosis and treatment. Traditionally supportive care programmes for TYA cancer patients and survivors tend to focus primarily on psychosocial issues such as well-being, relationships, education and finance [10]. Future supportive care interventions for TYA cancer patients should look to incorporate health behaviour promotion alongside these topics as a means to facilitate self-management and good health [20].

Akin to existing reports, participants within this study described experiencing improvements in physical fitness as rewarding and motivating [21, 22]. Specifically, many participants described a reduction in fatigue and the benefit the program had upon their energy levels. This was to be expected given the strong evidence base which demonstrates physical activity is beneficial for the management of cancer-related fatigue during and after treatment [23]. Among adolescents and young adults, there is growing evidence that the same holds true [24–27]. For example, in one study of adolescent cancer survivors, exercise was found to partially mediate the relationship between fatigue and quality of life during treatment and the relationship between sleep quality and quality of life post-treatment [28]. However, whilst physical activity is consistently found to reduce cancer-related fatigue; fatigue has been identified as one of the primary barriers to engaging in exercise [29–31]. Cancer-related changes in appearance, feeling frail and body image are also barriers to engaging in exercise programmes. Participants within this study highlighted the benefit of the RENEW programme being individually tailored allowing opportunity for the programme to be adapted to personal needs/capabilities. The individualised approach to exercise prescription taken within the RENEW programme is reflective of the current American College of Sports Medicine guidelines which advocate tailored exercise prescription which accounts for any contraindications and personal level psychosocial and physical barriers to exercise [6].

The one-to-one personal support provided within the RENEW programme appeared specifically beneficial at developing participants confidence and motivation. Many participants highlighted that they felt reassured by the advice provided by their L4 personal trainer. This is unsurprising given the well-documented uncertainty and worry which often accompanies cancer survivorship at a young age [32]. Similar to studies carried out among adult cancer survivors, participants within this study described the importance of the L4 instructor tailoring the programme to their ability but also challenging them to progress to higher levels of exercise intensity [21, 33]. There is concern however that instructor led programs may encourage cancer survivors to become dependent on the support provided and limit their ability to exercise in ‘real-life’ settings outwith of formal interventions [19]. Community-based sport programmes which provide less structured exercise such as football and basketball groups have been suggested as alternatives to gym-based structured interventions for young adult cancer survivors [34, 35]. Future research should look to evaluate whether these forms of intervention provide sufficiently tailored physical activity support and whether these forms of intervention result in improved/better long-term maintenance of physical activity behaviour change.

The ease of access to the RENEW programme was highlighted by participants as a key element of the programme. This is contrast to patient experience of traditional exercise interventions which are typically delivered in a university or hospital research setting, involve stringent eligibility criteria and provide no participant autonomy on the amount or type of exercise undertaken during the intervention period. Specifically, participants praised the referral process facilitated by Trekstock. Participants also highlighted the benefit of having unlimited gym access and access to the variety of classes at the gym. However, some noted that travel to and from central London was onerous and financially expensive. Distance has previously been identified as a predictor of adherence to exercise-based interventions [36, 37]. Some young adults within the study also spoke of feeling isolated and self-conscious when using the gym facilities outside of the personal training sessions with the L4 personal trainer. Previous studies have demonstrated that social support is positively related to changes in physical activity among young adult cancer survivors [38]. The inclusion of a buddy system or a group-based format of delivery could support young adult cancer survivors to remain motivated and help normalise the experience of taking part in a formal exercise programme [39]. Facebook has been successfully used in the past to support young adult cancer survivors to connect and provide peer support and encouragement to remain active (FITNET) [39]. In addition, feedback and opportunities for long-term follow-up may be beneficial at supporting young people with cancer to sustain physical activity behaviour change beyond the completion of formal exercise support programmes.

### Strengths and limitations

This study provides insight on the factors influencing the participation and engagement with a community-based exercise programme delivered by a charitable organisation. This is novel and reflects the shift in exercise-oncology research from small pilot effectiveness trials towards pragmatic evaluations of real-world interventions [40]. The use of semi-structured interviews to collect the data for this study was highly valuable as it allowed exploration of ideas and collection of subjective data to supplement the quantitative data previously reported [13]. However, it should be acknowledged that sample described here (and within the quantitative report) is predominantly female and white British. This limits the generalisability of the results to the wider young adult cancer survivor population. However, the sample size if reflective of sample sizes reported within other studies of young adult cancer survivors [41]. Furthermore, it is possible that those with high interest in health and exercise agreed to participate in the RENEW programme and subsequent evaluation suggesting a degree of response bias.

## Conclusions

Young adult cancer survivors highlight that the cancer-specific, individualised and cost-free aspects of the RENEW programme helped them overcome barriers relating to side effects of cancer treatment and self-efficacy to exercise. These findings are informative for the further development and evaluation of supportive cancer programmes delivered by charitable organisations. Future work should look to identify if such programmes can be easily replicated in other geographical regions and if peer-support and follow-up advice sessions could be incorporated into the delivery.

## Electronic supplementary material

ESM 1 (DOCX 19 kb)

## Data Availability

All data and material is available on request.
