# Supplementary material for: Young adult cancer survivors’ experience of taking part in a 12-week exercise referral programme: a qualitative study of the Trekstock RENEW initiative
Source: Support Care Cancer. 2020 Sep 22;29(5):2613–20. doi: 10.1007/s00520-020-05746-w (PMC7981325; doi:10.1007/s00520-020-05746-w)
Supplement: Supplementary file 1 — (DOCX 19 kb) [file 520_2020_5746_MOESM1_ESM.docx]

**Supplementary File A**

Semi-Structured Telephone Interview Guide

Young adult cancer survivors’ (YACS) views on the RENEW programme

**Aims**:

- To evaluate YACS experience of the RENEW exercise programme
- To identify YACS preferred aspects of the programme and ideas for the future of the programme

Introduction

- Thank participant for taking the time to participate.
- Can you tell me a bit about yourself?

| Gender |  |
| --- | --- |
| Age |  |
| Employment |  |
| Cancer type |  |
| Treatment status |  |
| Did you complete the RENEW programme or stop before 12-weeks? |  |
| When did you complete (/stop) the programme? |  |

- Summarise the purpose of the interview – *We’re evaluating the RENEW programme and your views are really valuable*. *I’m going to ask you a few questions about what motivated you to participate and how you found the programme.*
- Consent – *The interview should take about 20 minutes. Everything you say will be completely confidential and the data collected will be anonymised. Are you happy to continue?*

1. Motives and expectations for taking part.

- Why did you sign up to the programme? (*someone else said to e.g. doctor or family member / wanted to get fitter / wanted to do something to improve effects of cancer and its treatment e.g. fatigue / wanted to do something to improve cancer outcomes / have enjoyed previous events with Trekstock so decided to give it a go / something to do to get out of the house…) – explore their reasons*
- How did you hear about the RENEW programme? *(healthcare professional / Trekstock / word of mouth)*
- How important is exercise to you? *(do you consider exercise an important part of your lifestyle, that you try to dedicate time to? Do you know about the benefits of exercise for health?)* Why?
- Before you started the programme what were your expectations? *(did you understand the structure of the programme i.e. 12-week programme with online support, weekly check-ins and monthly one-to-one sessions?)*
- What were you hoping to get out of the programme? *(visible results i.e. noticeable improvement in fitness or symptoms / confidence to exercise e.g. knowing how to navigate the gym / exercise plan to be able to continue) What changes were you expecting to see?*

1. Whether the programme met expectations.

- Did the programme meet your expectations? *(e.g. if hoping for noticeable results did you see the differences you were hoping to see? / if hoping for confidence to exercise do you now feel able to use the gym and exercise without the PT?*
- Was the programme what you thought it was going to be? *(was there anything missing from the programme that you had expected/ hoped for?*

1. Satisfaction with the programme.

- How was your experience of the programme overall? *(Were you satisfied with the programme?* *Did you have a positive experience? Did you enjoy the programme? Are you glad you participated in RENEW? Would you recommend the programme to other cancer survivors?)*
- What have you gained from the programme? *(what effects has it had on your life in general as well as your fitness and physical activity?)*

1. Challenges faced during the programme.

- Did you face any challenges during the programme? *(barriers to participation or adherence e.g. difficulty getting to the gym / sticking to the programme because of fatigue or other symptoms / maintaining motivation / finding time to fit the programme into your life?)*
- If participant stopped before completing the programme 🡪 why were you unable to complete the programme? *(reason + explanation)*
- Did you face any barriers when enrolling in the programme? *(were there any difficulties in signing up to RENEW?)*

1. Ideas for the future of the programme

- Do you have any ideas for the future of the programme? *(e.g. ideas for different activities / how more help could have been offered)*
- Was there anything you would have liked to have been different about the programme? *(e.g. more group support / more online support …)*
- Would you have signed up if the programme was based around group sessions?
